# An inevitably ageing world: analysis on the evolutionary pattern of age structure in 200 countries

**DOI:** 10.1098/rsos.241988

**Published:** 2025-04-04

**Authors:** Jiajun Ma, Qinghua Chen, Xiaosong Chen, Jingfang Fan, Xiaomeng Li

**Affiliations:** ^1^ School of Systems Science, Beijing Normal University, Beijing, People’s Republic of China

**Keywords:** population dynamics, ageing, demographic dividend, demographic transition, tensor decomposition

## Abstract

Human reproductive, mortality and migration behaviours can often be standardized across countries. However, the universality of population growth laws remains a subject of debate. This study models age-specific population data as a three-dimensional tensor and applies high-dimensional tensor decomposition to uncover macro-level patterns in demographic systems across 200 countries over the past 70 years. The findings reveal that, while disparities in age demographics are widening, most nations follow remarkably similar evolutionary trajectories, differing mainly in the pace of change. A universal transition from the demographic dividend to population ageing is evident, with even labour-abundant regions such as Africa, Asia and South America inevitably facing this demographic shift. By incorporating economic indicators, the study quantitatively demonstrates the coordination between population structure and economic growth, while identifying notable exceptions, such as Gulf states that remain both affluent and youthful, and countries like North Korea, Tunisia, Sri Lanka and Ukraine that are ageing without first achieving significant economic wealth.

## 1. Introduction

After World War II, ‘peace and development’ emerged as central themes for most countries and regions. Concurrently, the global population expanded dramatically, growing from 2.5 billion in 1950 to 8 billion in 2022. According to United Nations projections, this growth is expected to continue, reaching approximately 8.5 billion by 2030, 9.7 billion by 2050 and 10.4 billion by 2100 [1]. Humans occupy a dual role as both producers and creators of resources, as well as consumers and destroyers of the same. The rapid pace of population growth, coupled with vigorous urbanization, has intensified pressures on natural environments and resources [2]. At the same time, as a key driver of production and the primary medium for societal and cultural development, population serves as a fundamental pillar for national stability and cultural continuity. Consequently, related studies on fertility, mortality, migration and ageing of the population have always been the concern of researchers and governments [3–5], forming the basis for inquiries across diverse fields such as economics, trade, environmental science and political science [6].

The age structure of a population shapes not only the potential for future growth within specific age groups but also the trajectory of the overall population. Significant variations exist in mortality rates, life expectancy and inequality across different age groups. As a result, understanding the heterogeneity and evolution of age structures is as crucial as analysing total population figures [7–9]. Age-specific population data form a complex three-dimensional tensor comprising time, country and age group. However, owing to the lack of robust systematic analytical methods, existing literature often relies on summing, averaging or subtracting data across one or two dimensions. Such reliance on statistical averages or expected values risks overlooking critical transitions in age-specific demographic trends, such as failing to detect the reversed relationship between life-span variation and life expectancy among different cohorts [10–12]. Additionally, many studies focus on specific indicators for particular regions, often neglecting national or structural details. This approach limits cross-country comparisons and impedes the identification of universal patterns in the dynamic evolution of age structures [5,13]. For example, research on Africa and Europe often prioritizes vastly different demographic challenges, reflecting the unique issues faced by each region. This regional specificity further complicates efforts to uncover overarching global trends in population ageing.

Empirical research has revealed patterns of convergence in age structure evolution worldwide [10]. Based on these observations, demographers have developed mortality transition models [14,15] that describe demographic transitions driven by factors such as advances in epidemiology [16], medical advancements, behavioural habits [17], economic status and even social security [18]. These foundational studies underscore both the existence of mortality transitions and their multifaceted drivers, providing a critical framework for analysing age structure evolution across countries. Mortality transition models are now widely applied in long-term population forecasting [19,20]. Researchers have found that, regardless of geographical location, economic status or cultural background, human lifecycles and age-specific mortality follow remarkably consistent patterns [21,22]. Similarly, human reproductive and migration behaviours exhibit notable regularities [23], with corresponding models demonstrating strong predictive accuracy [24–26]. Given these similarities in human behavioural patterns, we are prompted to explore whether a universal law of population growth may be embedded within the vast yet and unstructured demographic data collected from diverse countries.

While significant progress has been made in understanding mortality transitions, migration patterns and fertility predictions, few studies have successfully integrated these aspects to analyse the universal patterns of human age structure evolution through comparative cross-country studies. In the twenty-first century, there have been two major underlying demographic shifts, ageing and urbanization, which are also drivers of significant social transformation [27–29]. With declining mortality rates and widening disparities in life expectancy among the elderly across more countries [20], a critical question arises: ‘is aging an inevitable trend (following demographic dividend) that no country can avoid, even if measures are taken to counteract it?’ To address this, it is crucial not only to examine regions where ageing is already evident, but also to explore the fundamental trajectory of age structure evolution through high-dimensional data analysis.

It is challenging to analyse high-dimensional tensor data without compressing information. Eigen microstate theory, originating from statistical physics, is used to analyse the macroscopic behaviours of a system composed of multiple interacting objects [30,31] and has played a role in the analysis of different complex systems in nature and economic society [32,33]. Unlike traditional statistical methods, the eigen microstate (EM) does not reduce the dimension of high-dimensional tensors, but instead, explores some macro phenomena and evolution laws of the system and the collective behaviours of objects by integrating information from different dimensions [34]. Examples include the prediction of El Niño and La Niña events from temperature changes at 18 048 observation stations and the description of the coevolution mechanism of the energy or material sector from price fluctuation of 1600 stocks [32]. This approach can similarly be used to analyse the evolution of complex systems represented by age-specific population data across 200 countries, offering an alternative to existing approaches that rely on regional aggregation, statistical analysis or isolated examination of individual factors such as fertility, mortality and migration.

The remainder of this article is organized as follows: §2 outlines the database and methodologies employed for tensor analysis and higher-order decomposition. This approach reduces the complexity of the original empirical data while capturing the macroscopic behaviours, evolutionary patterns of the overall system and individual deviations. Then §3 demonstrates the decomposition results of comprehensive age structure data, aligning with findings from traditional statistical methods and analyses of specific factors, but also offers a more precise and holistic interpretation of population age structure evolution across national, age structure and temporal dimensions. The analysis quantitatively illustrates a universal trend in the shifting proportions of elderly and working-age populations, indicating that the demographics on all continents seem to be inevitably shifting towards an ageing population, suggesting that all the continents, including currently ‘young’ regions like Africa, Asia and South America with their ‘demographic dividends’, are inevitably moving towards ageing populations. Additionally, by integrating economic development indicators, we gain deeper insights into overarching patterns of population structure change and the practical challenges encountered by specific cases, such as countries experiencing ageing before achieving economic prosperity. Finally, §4 concludes the paper with a discussion of the findings and their broader implications.

## 2. Data and methods

### 2.1. Data source

The world population data were obtained from the United Nations Department of Economic and Social Affairs, as World Population Prospects 2022 (WPP2022), including overall and age-specific population. The data covered 200 countries/regions^1^ and was divided into 21 age groups. These annual population data were divided into two parts: the data from 1950 to 2021 were estimated by the United Nations, and the data from 2022 to 2050 are predicted values.

Despite the limitations of the WPP2022 database, such as the use of model life tables which can lead to reduced accuracy in mortality indicators, particularly in African countries, it remains a valuable resource for demographic data owing to its comprehensive mortality data across countries [10]. The database’s estimation methods are generally scientific, and for the purposes of this study, which considers age structure as a whole without distinguishing between fertility and mortality rates, using the WPP database is still appropriate. The data from WPP2022 can provide a broad overview for age structure evolution.

The WPP2022 includes 10 deterministic projection scenarios in terms of assumptions for fertility, mortality and international migration. The data selected are in the first scenario, based on annual estimates and standard projection scenarios. For fertility and mortality, the analysis uses medium assumptions, based on median probabilistic projection. For international migration, it follows the medium projection.

To show the differences in the evolution of age structure among countries with different income levels, we divided 200 countries into four categories according to the standards of the World Bank, including 28 low-income, 54 lower-middle-income, 48 upper-middle-income and 63 high-income countries, in addition to seven countries or territories where the economic situation is unknown. The details of the dataare provided in table 1.

**Table 1.** Data description. (America etc mentioned in the passage refers to Oceania, North America and South America.)

variable	variable description	data source
population by age group	quinquennial population by 1-year age groups—both sexes. De facto population as of 1 July of the year indicated classified by 1-year age groups (0−4, 5−9, 10−14, …, 95−99, 100+). Data are presented in thousands. Total population (both sexes combined) by 5-year age group, region, subregion and country, annually for 1950−2021 (thousands). Estimates, annually for 1950−2021 (thousands). Medium fertility variant. This dataset is licensed under Creative Commons license CC BY 3.0. The list of countries and continents are in table 3 (appendix H)	https://population.un.org/wpp/downloads?folder=Probabilistic%20Projections7group=Population
country economic classification	data were from the World Bank, which divides countries into four categories according to their economic level: low-income economies, lower-middle-income economies, upper-middle-income economies and high-income economies. This dataset is licensed under Creative Commons Attribution 4.0	https://datahelpdesk.worldbank.org/knowledgebase/articles/906519-world-bank-country-and-lending-groups
map template (.shp)	the map template is from WORLD Country Polygons in the World Bank. This dataset is licensed under Creative Commons Attribution 4.0	https://datacatalog.worldbank.org/search/dataset/0038272

### 2.2. Tensor decomposition and eigen microstates

The global population is a complex system that evolves with time and is composed of 200 countries, which are the agents of the system. Using the age groups i=1,2,...,M and the times t=1,2,...,L in sequence, we obtain the state series $S_{n}(i,t)$ of agent n , with n=1,2,...,N . In the literature, data analysis using EM theory usually focuses on two-dimensional matrices [32,34]. To describe the system state more comprehensively, we extended the data from matrix to tensors and used canonical polyadic (CP) decomposition [35,36] to obtain vectors describing different dimensions. CP decomposition is a method of decomposing a tensor into a sum of rank-1 tensors.

**Figure 1.**
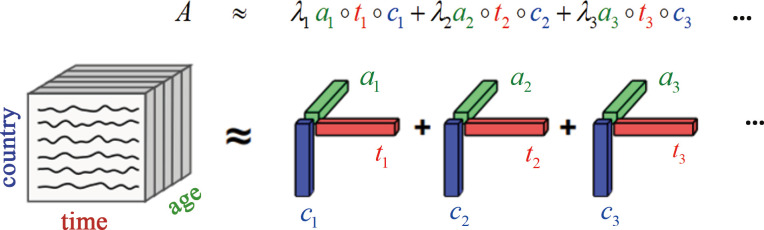
Canonical polyadic (CP) decomposition. The tensor 𝑨 is decomposed into a sum of rank-1 tensors, as ${𝒂}_{1}$ , ${𝒕}_{1}$ , ${𝒄}_{1}$ , ${𝒂}_{2}$ , ${𝒕}_{2}$ , ${𝒄}_{2}$ , ${𝒂}_{3}$ , ${𝒕}_{3}$ , ${𝒄}_{3}$ .


Here, we have M×L×N tensor 𝑺 , and $S_{n}(:,t)$ represents the age structure proportion of country n in year t . Then, normalize tensor 𝑺 from equation A=S/norm(S) , where $norm(S)=\sqrt{\sum_{i,t,n}S_{n}(i,t{)}^{2}}$ . As shown in figure 1, according to the CP decomposition and r=1,2,3...,R , the tensor 𝑨 can be factorized as:

(2.1) $$\begin{array}{lcr} & \begin{array}{rl} 𝑨 & \approx \sum_{r=1}^{R}\lambda_{r}{𝒂}_{𝒓}\circ {𝒕}_{𝒓}\circ {𝒄}_{𝒓}\\ A_{itn} & \approx \sum_{r=1}^{R}\lambda_{r}a_{r}(i)t_{r}(t)c_{r}(n). \end{array} & \end{array}$$

${𝒂}_{𝒓}\circ {𝒄}_{𝒓}$ constitutes the r -th eigen microstate (EMr) of the N agent system and ${𝒕}_{𝒓}$ describes the evolution of EMr over time. Figure 1 shows that it reproduces complicated empirical data with less information and fewer variables. ${𝒂}_{𝒓}$ represents the eigenvectors in the age structure dimension, ${𝒕}_{𝒓}$ represents eigenvectors in the time dimension and ${𝒄}_{𝒓}$ represents the eigenvectors in the country dimension. Then, the description of the tensor ( M×L×N ) is decomposed into two parts: one is the macroscopic behaviour of the whole system as ${𝒂}_{𝒓}$ ( M×R ) and ${𝒕}_{𝒓}$ ( L×R ), and the other is the quantification of each country ( N×R ). The principle of dimensionality reduction is to use less information to describe the original empirical data by identifying the macroscopic behaviour, evolution rules of the entire system and the difference in each agent’s compliance. Therefore, eigen microstate theory can help us find the basic evolution patterns of the overall population and structure in the world from complex population data, as well as the personalized characteristics of different countries.

Here, we used the alternating least squares algorithm to perform CP decomposition. After choosing the rank, we randomly initialized 500 times to avoid the local extremum and select the best-fitting solution described by the fit value. Then, we obtained the unique solution for a given rank. The fit value $f=1-norm(A-\hat{A})$ was loosely the proportion of the data described by the CP model, where $\hat{𝑨}$ is an approximate tensor. We used the Tensor toolbox (v. 3.4) of MATLAB to obtain CP decomposition results. We tested the robustness by comparing the results from 10 experiments, including CP decomposition, singular value decomposition (SVD) and high-order singular value decomposition (HOSVD) [35,37].

### 2.3. Age structure regression and coefficient analysis

The results of the previous decomposition describe the macromorphology of the world population system, with each eigenstate describing a characteristic of the system. When analysing the evolution pattern of different countries, the consistency of each country with these macro features and its evolution path need be quantified. Here, we standardize the age structure proportion $S_{n}(:,t)$ and describe it with R characteristics as ${𝒂}_{1}$ , ${𝒂}_{2}$ , ${𝒂}_{3}$ ,..., ${𝒂}_{𝑹}$ (the eigenvectors in the age structure dimension got by equation 2.1):


(2.2) $$S_{n}(:,t)=A_{n,t}*a_{1}+B_{n,t}*a_{2}+C_{n,t}*a_{3}\;+\;...$$

Parameter $A_{n,t}$ and $B_{n,t}$ and $C_{n,t}$ describe the impact of characteristics ${𝒂}_{𝒓}$ on the age structure of country n in year t .

## 3. Results

Based on empirical data, the tensor 𝑨 captures population evolution patterns across 200 countries, segmented by age group, spanning from 1950 to 2021. This M×L×N tensor is challenging to analyse quantitatively from a global and dynamic perspective using traditional methods. Previous studies often isolate individual factors, such as fertility, mortality, or migration, to investigate the patterns and drivers of population structure transitions, as in studies on changes in mortality patterns within the framework of demographic transition theory [12,14].

In this study, we aim to directly decompose the tensor 𝑨 across three dimensions—age structure, time and country, rather than empirically breaking it down into topics such as fertility, mortality and migration. First, we needed to determine the appropriate rank of decomposition. Since finding the rank of a tensor is NP-hard (Nondeterministic Polynomial-time hard) [35], we employed the fit value to guide the rank selection for the CP model, setting R=3 . We then conducted 10 experiments, each initialized randomly 500 times, and selected the final results based on fit values and orthogonality. The CP decomposition yielded three primary eigenmicrostates, which collectively explain 88.65% of the information in the data. This approach enabled us to extract evolution patterns of the global population system represented by ${𝒂}_{𝒓}$ (age), ${𝒕}_{𝒓}$ (time) and $c_{r}$ (country characteristics) for r=1,2,3 . The detailed analysis is described in the subsequent sections. We also applied alternative methods, including SVD and HOSVD decompositions, which produced comparable findings [35,37]. Further decomposition results and details are available in appendix A.

### 3.1. Evolution patterns

It is essential to recognize that population structure reflects a complex interaction of multiple factors. While the literature frequently focuses on individual factors, such as fertility, mortality or migration, to derive insights with practical applications, these targeted approaches may lack comprehensiveness. However, such focused approaches may fall short in terms of comprehensiveness. Our method addresses this limitation by directly analysing and decomposing the complex data on population structure, revealing three primary patterns. These patterns offer a broader perspective on the evolution of global population age structures, complementing and expanding upon the findings from studies that approach the topic from various perspectives. By integrating these diverse perspectives, our method provides a more comprehensive representation of the dynamics underlying the multifaceted evolution of population age structures.

#### 3.1.1. First eigen microstate

Following World War II, global population trends showed overall growth, though at a decelerating pace, largely owing to declining fertility rates [38,39]. The classic demographic transition theory outlines a shift from high birth and death rates to lower rates in both domains. However, by the end of the twentieth century, fertility rates in some European countries fell below replacement levels, marking the onset of ‘ultra-low fertility.’ To explain this, the literature introduced the ‘Second Demographic Transition Theory’ [38,40], which highlights the transformation of marriage, family, and reproductive patterns within a post-modern context.

**Figure 2.**
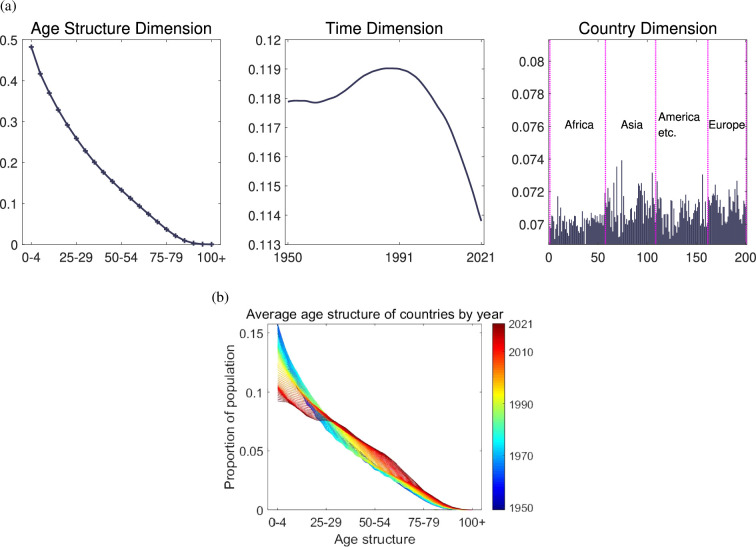
Continuous population growth and gradual slowdown in various countries. (a) Left: ${𝒂}_{1}$ has typical pyramid features with the population proportion decreasing with increasing age. (a) Middle: ${𝒕}_{1}$ reaches the maximum value in approximately 1991. (a) Right: All positive ${𝒄}_{1}$ means the first eigenstate is universal to all countries. (a) First eigen microstate is EM1. (b) Global population structure and comprehensive indicators.

This study offers a more rigorous and precise quantitative description of the evolution of global population structure. Here the First eigen microstate (EM1) had positive ${𝒂}_{1}$ , ${𝒕}_{1}$ and ${𝒄}_{1}$ (figure 2a). ${𝒄}_{1}$ for all countries was located in [0.0688, 0.0739], its variance was $7.9121e^{-07}$ and the gap between countries was small, indicating that the first eigenstate is universal to all countries in the world for more than 70 years after World War II (figure 2a, right).

For the age structure, ${𝒂}_{1}$ has typical pyramid features with the population proportion decreasing with increasing age. The downward convex curve is typically associated with a population growth pattern characterized by high fertility and low ageing (figure 2a, left) [41]. The value range of ${𝒕}_{1}$ was [0.1138, 0.1730]. This growth type of age structure as ${𝒂}_{1}$ was generally present for 72 years, and its influence increased first and then decreased, with ${𝒕}_{1}$ reaching its maximum value in approximately 1991 (figure 2a, middle). Figure 2b describes changes in the average age structure of all countries, from the 1950s in blue, accompanied by declining fertility rates, to the 1990s in green. It shows little change in age structure before the 1990s and then deviates rapidly from the age structure described by ${𝒂}_{1}$ , showing a relative ageing prominence and reduction in fertility (in red). This method provides more precise quantitative evidence for existing research conclusions across three dimensions: age, time and country. It represents the trend of maintaining population growth in most countries, but the growth is gradually slowing down.

#### 3.1.2. Second and third eigen microstates

In addition to the total population and growth rate, changes in age structure, particularly shifts in the working-age population, are also of significant concern. The emergence and decline of the labour force dividend resulting from demographic shifts is a central issue in this field. Researchers have noted that countries often follow a common trajectory, progressing from the onset of the demographic dividend to its eventual decline [42,43]. This pattern is evident in the findings of this study.

**Figure 3.**
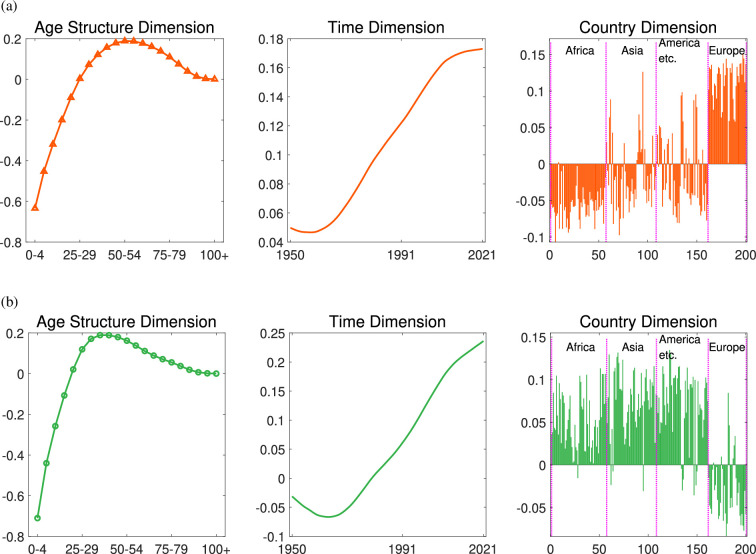
Evolutionary characteristics of the elderly and labour increasing. (a) For EM2, the largest age group is aged 50–54 (left), which is obvious in Europe and could be considered to describe the growth of the elderly population. (b) For EM3, the largest age group is aged 35–39 (left), describing the growth of the labour force structure.

Here, the second and third eigen microstates (EM2 and EM3) describe two types of evolutionary trends (figure 3). For the countries, ${𝒄}_{2}$ and ${𝒄}_{3}$ had both positive and negative values. Most countries had significant values of ${𝒄}_{2}$ and ${𝒄}_{3}$ , meaning that these two trends together characterize the evolution of almost all countries. The countries corresponding to the positive value of ${𝒄}_{2}$ are mainly concentrated in Europe, and the countries corresponding to the positive value of ${𝒄}_{3}$ are mainly concentrated in Africa, Asia and other regions. We draw the specific values in the map in appendix B. Existing research on working-age or ageing populations commonly defines the working age as individuals between 15 and 64 years [44,45], or between 20 and 65 years [46], while ageing is often defined as those aged 60 years or older, or 65 and older [47]. These definitions are typically arbitrary or based on empirical conventions [48]. In this study, however, the characteristics of age structure are derived directly from the data, capturing these dimensions without reliance on predefined age ranges. Here, the age structure showed common features of ${𝒂}_{2}$ and ${𝒂}_{3}$ , that is, the phenomenon of declining birth rate and an insufficient number of teenagers. However, the specific shapes of the two age structures were different. For EM2, in terms of proportion of the population, the largest age group is aged 50–54, and the older population structure generally showed a significant increase (i.e. thick-tailed characteristics), so it could be considered to describe the obvious growth of the elderly population (figure 3a). By contrast, for EM3, the largest age group is aged 35–39. Moreover, it was no longer evident before the age of 20 and after the age of 60; thus, we believe that EM3 describes the growth of the labour force structure (figure 3b). While existing statistical analyses highlight shifts in the ratio of the defined working-age population and defined elderly population, actual data offers a clearer view of the specific age ranges experiencing the most significant changes and their temporal evolution characteristics.

This approach provides a quantitative description of the transition ‘from demographic dividend to demographic drag’ [49,50] and enables a comparative analysis across different time periods. For the evolutionary behaviour, although ${𝒕}_{2}$ and ${𝒕}_{3}$ both exhibited a trend of decreasing at first and then increasing, ${𝒕}_{2}$ was always greater than zero and ${𝒕}_{3}$ fell below zero between 1950 and 1979. During this time period, there could be a transient opposite evolutionary trend, such as sufficient numbers of newborns and adolescents. By comparing the slope of the curve, we found that the enhancement rate of EM2 was relatively slower than that of EM3. The two phenomena of increases in the elderly and working-age populations will be analysed in detail in the following sections.

### 3.2. Several laws of population age structure evolution

Moreover, when comparing different countries, some researchers have noted an increase in age structure differences among nations [27,51]. However, others have observed a universal pattern in mortality changes, noting that while global convergence may not occur in a strict sense, groups of countries do follow very similar mortality change processes [10,17]. These findings are not contradictory; rather, they reflect the diverse trends shown by the composite metrics of population age structure across different periods and from varying perspectives. We propose that the evolutionary patterns of age structures, from abundant labour to ageing, are broadly similar across countries, although the sequence of these patterns’ evolution is gradually diverging among nations.

#### 3.2.1. The bifurcation of age demographics across countries

For country n , we regressed the standardized age structure proportions in year t with equation (2.2), where $B_{n,t}$ and $C_{n,t}$ describe the coefficient of the elderly and labour increasing. The range of the regression result determination coefficient $R^{2}$ was [0.6408, 1), with a mean value of 0.97763. In addition, 99.55% of $R^{2}$ values were greater than 0.80, 97.60% were greater than 0.90 and 87.78% were greater than 0.95. Here, we describe the age structure of countries at different times using three features: ${𝒂}_{1}$ , ${𝒂}_{2}$ and ${𝒂}_{3}$ .

**Figure 4.**
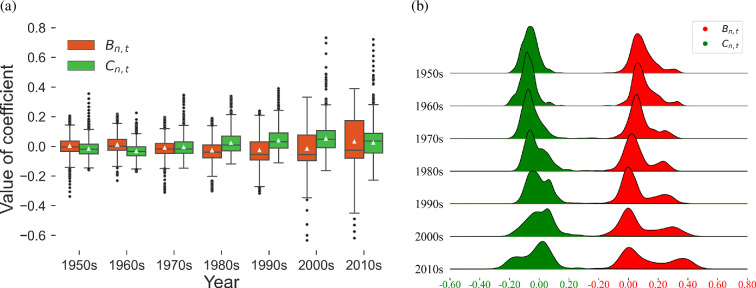
Distribution of regression coefficients. $B_{n,t}$ is the coefficient of EM2, which describes the growth of the elderly population, and $C_{n,t}$ is the coefficient of EM3, which describes the growth of the labour force structure. The dispersion of values for $B_{n,t}$ and $C_{n,t}$ in the boxplot has increased (a), with their distribution shifting from a unimodal to a bimodal pattern (b). This reflects a polarization in the age structure of countries.

In figure 4a, the red boxes represent the distribution of $B_{n,t}$ , and the green boxes represent the distribution of $C_{n,t}$ in year t , which describe the influence of elderly and working-age population growth on the age structure in different countries. In each box, the horizontal line from bottom to top represents the minimum, the first quartile, the third quartile and the maximum value, and the white triangle represents the mean value. First, the variances of $B_{n,t}$ and $C_{n,t}$ exhibited a gradually increasing trend, indicating that the differences in population structure between countries continued to grow, and the prominence of the elderly and working-age populations differed among countries. In traditional time-series analyses focused on age-group population ratios, it has been confirmed that differences in the working-age ratio and elderly ratio across countries are gradually widening [52,53]. Second, the mean of $B_{n,t}$ was initially greater than $C_{n,t}$ , and since the 1980s, the mean of $C_{n,t}$ has exceeded $B_{n,t}$ , indicating that the change in labour structure in many countries during this period was more significant, exceeding the trend of ageing. However, in the last decade, there have been many large positive values for $B_{n,t}$ , meaning that the structural characteristics of elderly individuals in many countries have been more significant.

The ridgeplot shows the distribution and evolution of $B_{n,t}$ and $C_{n,t}$ in detail (figure 4b). The distribution of $B_{n,t}$ and $C_{n,t}$ gradually evolved from a unimodal to a bimodal distribution, reflecting the trend of countries’ demographic characteristics from similarity to polarized, and the polarization of ageing has become more obvious in recent years. For $C_{n,t}$ , in the early years, the only peak was located in the area less than 0. In the 1980s, a positive subpeak appeared, and then it gradually increased and replaced the original negative peak to form a new maximum peak in the 2010s. The evolution of $B_{n,t}$ from a unimodal to a bimodal distribution was more pronounced. Although the current positive subpeak did not exceed the negative subpeak, it continued to increase and shift to the right side, indicating that the number of countries with an ageing trend is increasing, and the influence of the elderly is also strengthening. In addition, we plotted the ridgeplot from 2020s to 2040s (appendix C), and it shows that the peak with positive values surpasses the peak with negative values (figure 15).

#### 3.2.2. Alternating of the elderly and working-age population

**Figure 5.**
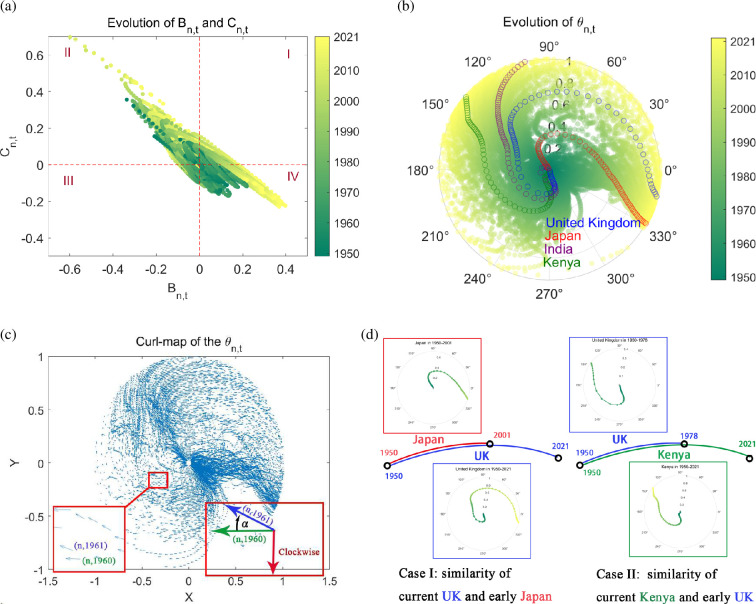
The alternating law of $B_{n,t}$ and $C_{n,t}$ . Despite growing disparities among countries, their developmental trajectories remain analogous, albeit at varying paces. (a) In rectangular coordinates. (b) In polar coordinates. (c) Evolution direction of $\theta_{n,\;t}$ . (d) Similar evolutionary paths. (*n, t*) is the vector from (0,0) to (*B_n,t_, C_n,t_*)

Figure 5a shows the evolution of $B_{n,t}$ and $C_{n,t}$ , and the indicators of each country during the period from 1950 to 2021 are represented by a series of dots from green to yellow (figure 5a). Taking the origin (0,0) as the centre, we found that the time-series dots of many countries rotated clockwise around the centre, that is, starting from the fourth or third quadrant, passing through the second and first quadrant, and finally returning back to the fourth quadrant. We analyse the meaning according to the characteristics of the quadrant coefficient. The third quadrant has abundant newborns and adolescents. In this quadrant, the coefficient of the labour force and the coefficient of the elderly population are both negative. The second quadrant has prominent labour force, where the labour force coefficient is positive and the elderly coefficient is negative. The fourth quadrant has the prominent elderly population, with the positive elderly coefficient and negative labour force coefficient. The first quadrant is the transitional period from prominent labour force to prominent elderly population. The most representative countries in each quadrant and their age structures are in appendix D. Most evolution paths coincided with this clockwise route, with different countries having various starting and ending regions.

Combining the theories of the two demographic transitions [16,18] with the recent mortality transition theory [17,19], the literature has identified a common pattern across many countries and regions: the shift from demographic dividend to ageing. Here this rule is more significant in polar coordinates. Figure 5 focuses on the angle in polar coordinates as $\theta_{n,t}$ , and the radius $r_{n,t}$ was uniformly quantified as an equidistant growth over time. Then, the evolution path of each country is uniformly close to the outermost circle r=1 over time. It illustrates the evolutionary trajectories of four nations, with Kenya represented in green, India in purple, Japan in red and the UK in blue.

Here, the characteristics of clockwise evolution are more pronounced. We used the vector field to analyse the evolution paths. The coordinate positions of two consecutive years form two vectors, and we calculated the cross product of the vector sequence. For example, the vectors of country n in 1960 and 1961 were selected, and the angle α between vector (n,1960) and vector (n,1961) can be determined by the direction of their cross product. If the cross product is negative, α is clockwise, while if it is positive, α is counterclockwise (figure 5c). The original data depicted in the box plot are illustrated by the circular points within figure 21). It shows since the 1990s, more than 70% of countries have experienced clockwise evolution, accounting for more than 80% of the paths. In the last 10 years, this clockwise ratio has been very close to 100%.

Although the disparities in age demographics among countries are increasing, many nations have strikingly similar trajectories in the transition between $B_{n,t}$ and $C_{n,t}$ , with the primary distinction being the pace of their evolution. Figure 5d illustrates the examples from three countries. Kenya’s age structure, which is currently dominated by the working-age population, closely mirrors that of the UK prior to 1978. The UK has since advanced into an ageing phase, and its evolutionary path bears a strong resemblance to Japan’s in 2001.

#### 3.2.3. Connection between age structure and economy

**Figure 6.**
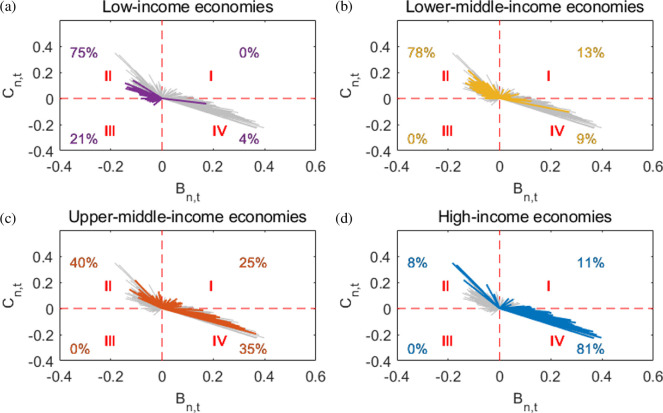
Characteristics of countries with different income levels. Quadrant II features a robust labour force with a positive coefficient $C_{n,t}$ , contrasting with a negative coefficient for the elderly $B_{n,t}$ . Quadrant IV is dominated by the elderly with a positive coefficient, against a negative labour force coefficient. Quadrants I and III act as transitional zones. The grey lines represent all countries. Specifically, (a) low-income economies are indicated in purple, (b) low-middle-income economies in yellow, (c) upper-middle-income economies in orange, and (d) high-income economies in blue.

The World Bank categorizes countries into four income levels, as follows: high income, upper-middle income, lower-middle income and low income. Figure 6 shows $B_{n,t}$ and $C_{n,t}$ in the period from 2021 to 2022 for each country. The grey lines connect the (0,0) and the positions of countries from 2021 to 2022, and the blue, orange, yellow and purple lines represent countries in four income levels. It also shows the proportion of each category of countries located in the first to fourth quadrants.

Most low-income countries fell in the second quadrant, while a large number of high-income countries were in the fourth quadrant. The remaining two categories lie between high-income and low-income countries. For example, 78% of low-middle-income countries were in the second quadrant, but 13% turned clockwise to the first quadrant. For upper-middle-income countries, only 40% remained in the second quadrant, with 25% in the first quadrant and another 35% transferred to the fourth quadrant. This means that, currently, the characteristics of increasing labour structure tend to appear in relatively low-income countries, while in high-income countries, the growth trend of the elderly population is more significant.

Economic development has led to a decline in fertility rates through women’s empowerment, increased parenting costs, competition for economic efficiency in childbirth and cultural shifts in childbirth [54,55]. At the same time, the improvement of medical standards has reduced the mortality rate of the elderly population [11,20]. Most countries exhibit a statistical pattern where age structure is negatively correlated with economic level. However, there are also some exceptions among them.

Notably, Democartic People’s Republic of Korea (PRK), as a low-income country, and Tunisia (TUN), Lebanon (LBN), Sri Lanka (LKA), Samoa (WSM) and Ukraine (UKR), as lower-middle-income countries, are all located in the fourth quadrant and accompanied by a more prominent elderly population structure, which shows a phenomenon of ‘getting old before getting rich’. The insufficient productivity and increased social burden brought about by ageing will make the economic development of these countries encounter inevitable difficulties. In addition, only six countries (Somalia (SOM), Central African Republic (CAF), Chad (TCD), Democratic Republic of the Congo (COD), Mali (MLI) and Niger (NER)) are in the third quadrant in 2021, which means that both the labour force and ageing population in these countries are not prominent. For example, Niger (NER) is the world’s youngest country, with 49.0% of the population aged 0−14 in 2021 [56]. The age structures of these countries and some discussions are in appendix E.

There are five high-income countries still having a significantly increased proportion of the working population, including Bahrain (BHR), Oman (OMN), Qatar (QAT), Saudi Arabia (SAU) and United Arab Emirates (ARE), which are all Gulf countries near the Persian Gulf. These countries have rich oil and natural gas resources and high-income levels. The permanent foreign residents in Bahrain (BHR) and Oman (OMN) account for approximately 50% of the total population [57], while the United Arab Emirates (ARE) and Qatar (QAT) have more than 80% of the foreign population [58,59]. The continuous influx of labour population makes these countries continue to have a relatively sufficient working population.

### 3.3. Future evolutionary trajectory

The literature notes that population ageing is no longer confined to developed regions such as Europe; it is emerging as a global phenomenon, with countries in Asia and Latin America also experiencing increasingly older age structures [60,61]. Africa, meanwhile, remains the youngest continent, but whether it will also face ageing issues in the future is a question worth exploring quantitatively [62]. The complexity of age structure as a multidimensional vector poses challenges for comparative studies across time and countries. To date, no researcher has clearly delineated the dynamic relationship between changes in population age structure and economic growth. However, by reducing the dimensionality of age structure using indicators such as $B_{n,t}$ and $C_{n,t}$ , researchers aim to better compare various countries’ metrics and interpret general patterns in the coupled evolution of population and economic systems. Indeed, the question of whether Africa will also encounter ageing issues in the future is one that can now be explored quantitatively.

For most countries, the higher the income level is, the more obvious the characteristics of ageing, and accordingly, the characteristics of an abundant labour force are no longer significant. Since the evolution of the age structure has a certain continuity, if we do not consider the change in birth and death rates and ignore the impact of international migration, the abundance of the labour force in a certain period will naturally bring the ageing trend after a period of evolution. Therefore, is it inevitable for a country to experience a path from natural population growth to a prominent labour force structure and then to an ageing trend? If such a structural evolution pattern is inevitable, the world population will not continue to grow rapidly, as many researchers fear. Here, we use the prediction of the United Nations data, extend the time series of $B_{n,t}$ and $C_{n,t}$ and describe the evolution pattern of the regional age structure from 1950 to 2050.

**Figure 7.**
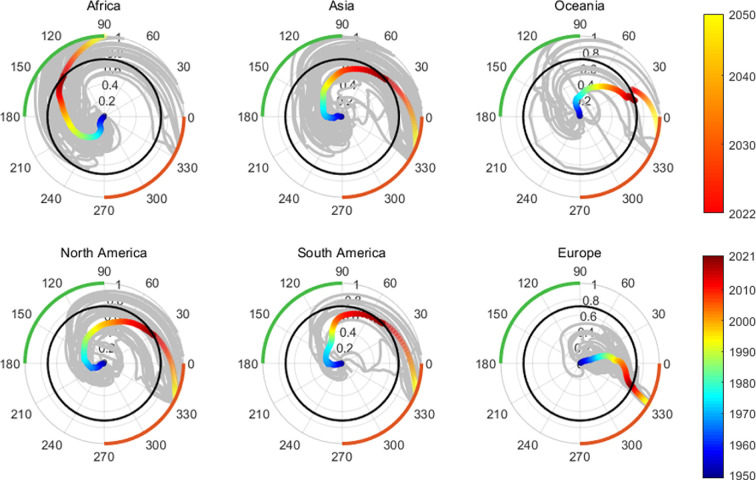
Evolution trend of the six continents. In polar coordinates, the outermost red sector corresponds to quadrant IV of the original Cartesian coordinate system, emphasizing the concept of ageing. By contrast, the green sector represents quadrant II, which highlights a robust labour force. The black circle serves as a demarcation line: within the circle, the evolutionary trajectory spans from 1950 to 2021, while beyond the circle, the projected path extends from 2022 to 2050.

Figure 7 shows the evolution trend of the age structure for the six main continents. For each continent, the grey lines are the evolution paths of all countries, and the coloured lines are the paths formed by their average values, where blue to red represent empirical data and red to yellow represent forecast data from the United Nations, with the black circle serving as the dividing line. Clockwise evolution patterns generally exist in all continents within a century. For Europe, which has entered the fourth quadrant, its population structure will continue to develop in the direction of ageing and labour shortages over the next 30 years. For Asia, Oceania, North America and South America, their age structure will gradually approach that of Europe, which will need face the problem of ageing at different times. For Asia [60,63,64], North [65] and South America [61,66], their speed of entering the fourth quadrant is significantly higher than Oceania, indicating that they will face the situation of ageing and labour shortage more quickly.

Currently, the age structure of Africa is still relatively young, and the working-age population is abundant. However, Africa has also shown a typical clockwise evolution trend; in addition, some African countries’ angular velocities are significantly higher than those in other regions. Therefore, for Africa, if there are no significant changes in birth, death and migration in the coming decades, its proportion of the working population will continue to decline [62,67] and its labour advantage will be negligible in 2050 ( $\theta_{n,t}\approx 90^{\circ }$ ). At that time, Africa is likely to face the challenges brought by the ageing trend, similar to Asia and South America a few years before. Although the total population of most countries has experienced continuous growth since World War II, by analysing the universal path of age structure evolution in various countries, we believe that most countries will go through the development from continuous growth to abundant labour force and eventually ageing, including the current ‘young’ Africa, and Asia, South America with ‘demographic dividend’. Even if some countries are not rich, they will still face the potential risk of insufficient population growth and ageing trends in the future.

**Figure 8.**
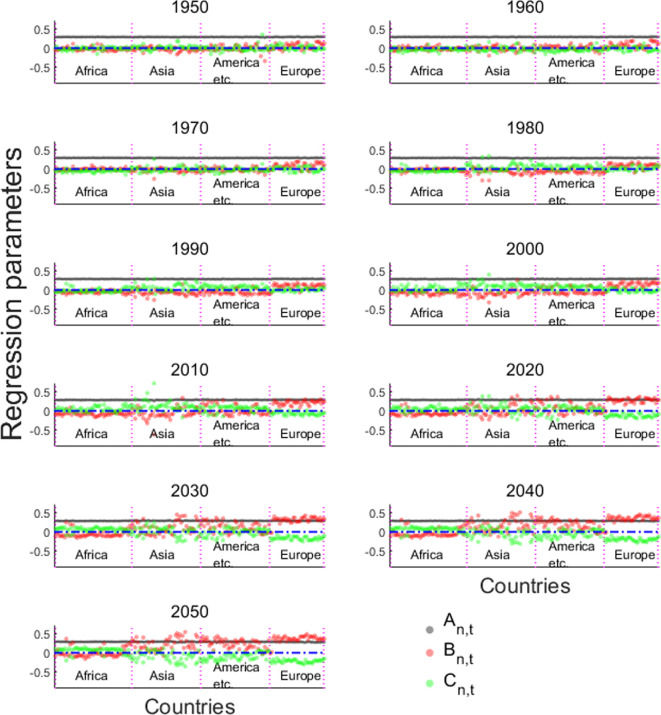
Evolution of three eigen microstates. $A_{n,t}$ , $B_{n,t}$ and $C_{n,t}$ are the coefficients of EM1, EM2 and EM3 for countries in different years. $A_{n,t}$ represents stability and consistency. Since the 1980s, many countries have experienced a surplus in the labour force, with $C_{n,t}>0$ . However, in the twenty-first century, this trend has reversed, with numerous countries now facing a decline in $C_{n,t}$ ( $C_{n,t}=0$ or even $C_{n,t}<0$ ). Europe began experiencing an ageing population, marked by $B_{n,t}>0$ , in the 1980s, and this trend later spread to Asia, America and other regions. By 2030, in some countries, $B_{n,t}$ is expected to surpass $A_{n,t}$ , becoming the dominant demographic characteristic.

**Figure 9.**
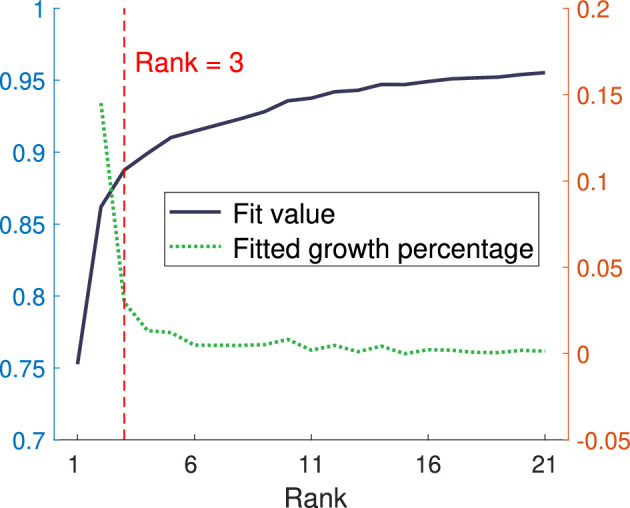
Rank selection based on fit value.

**Figure 15.**
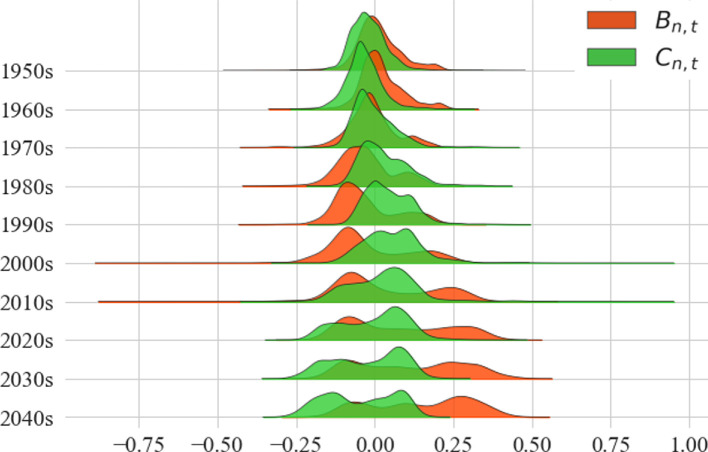
Ridge map with forecast data.

**Figure 21.**
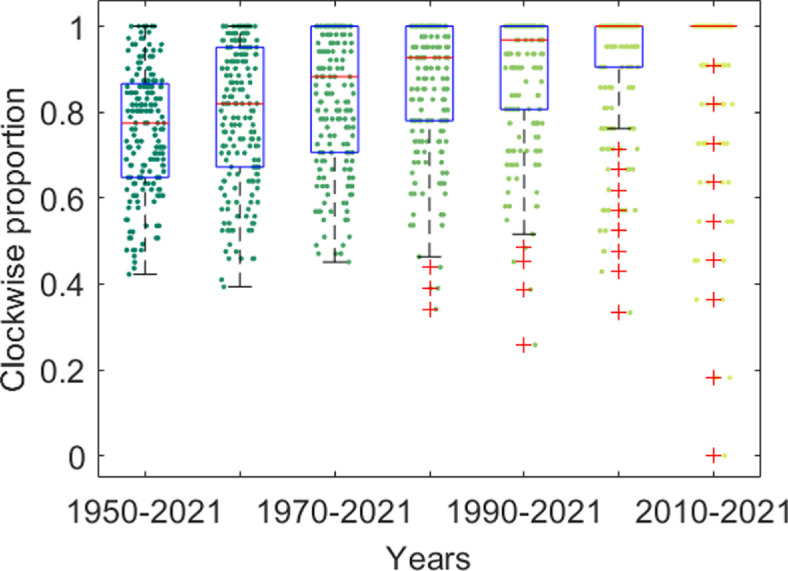
Clockwise evolution proportions, for analysing the alternating of the elderly and working-age population.

The time series of different countries can also indicate this one-way evolution trend (figure 8). At an interval of 10 years, we showed the evolution trend of the three age structure characteristics in various countries, as black for $A_{n,t}$ , red for $B_{n,t}$ and green for $C_{n,t}$ . After World War II, the characteristics of continuous population growth in most countries have not changed, which is reflected in the stability and consistency of $A_{n,t}$ . However, according to the forecast data, in the future, the characteristics of sustained growth in most European, some Asian, South American and Oceanian countries will weaken, reflecting a significant decrease in $A_{n,t}$ .

The characteristics of elderly growth were reflected as early as the 1950s–1970s. Later, with the increase in the working population, some American and Asian countries showed a negative $B_{n,t}$ , which means that the proportion of the elderly population has declined. From 1990 to 2020, a high proportion of the working population generally appeared in most regions except Europe, with a large number of positive $B_{n,t}$ . In addition to Europe, which has already shown the characteristics of ageing, most countries in Asia, South America, Oceania and even some African countries will begin to experience ageing trends after 2020. At the same time, most countries will face an insufficient labour force, with a large number of negative $C_{n,t}$ .

## 4. Conclusion

Declining fertility, increasing longevity and the progression of large cohorts to older ages are causing elder shares to rise throughout the world. People always tend to focus on areas where problems have occurred; for example, researchers have been concerned about ageing in Europe for decades, and in recent years, they have expanded the focus to Asia and Latin America, analysing the influence of population age structure on economy, consumption, medicine, environment, culture and even politics. While many governments are wrestling to encourage fertility and delay ageing, most African countries are still troubled by population explosion and the unemployment of young people, as in Asia several years ago. The United States census reported, page 6, ‘Africa is exceptionally young in 2015, and will remain so in the foreseeable future’ [68]. If we are no longer limited to the traditional method of data statistics but interpret the evolution of the age structure in the past 72 years, such an optimistic judgement is worth pondering.

This study suggests a complex system described by age-specific data from 200 countries during 1950−2021, which constitutes a complex tensor with three dimensions: time, country and age group. EM theory, originating from statistical physics, does not reduce the dimension of high-dimensional tensors as a traditional statistical method but explores some macro phenomena and evolution laws of the system and the collective behaviours of objects by integrating information from different dimensions. Here, age structure is the microstate of each country. The changes in these individual microstates over time could emerge from the macrostate evolution of the entire global population system.

First, it finds three main characteristics of global population evolution in the past 72 years and restores most information in the original age-specific data with three sets of eigenvectors; that is, 88.65% of the information described by 3 02 400 values is reproduced with just 879 values. It shows that after World War II, the population of most countries continued to grow, but the growth rates had different slowdowns (as EM1). Since the 1950s, the world has been evolving towards ageing.

In addition, the analysis including the microstate of each country will show more evolution laws in addition to the current ageing situation. The iteration of the population age structure has inertia, which presents the growth and coexistence of the working population and the elderly population. Here, it constructs a space composed of two macrostates, the prominent working population (EM3) and the prominent ageing population (EM2). Then, we could compare the microcosmic state of countries by their position in space at different times and describe the macroscopic evolution law of the whole system. Recently, 75% of low-income countries were located in the EM3 area, and 81% of high-income countries were located in the EM2 area. For the remaining two categories, 78% of low-middle-income countries and 40% of upper-middle-income countries were in the EM3 area, and 9% of low-middle-income countries and 35% of upper-middle-income countries were located in the EM2 area. It shows that with economic growth, the country’s population structure has a universal rule of transition from a sufficient labour force to an ageing population.

Finally, it draws the evolutionary path of countries in the two macrostate spaces, where most paths turn clockwise, and the prominence of the age structure ranges from the newborn population to the working population and then to the ageing population. The World Bank’s forecast data extended these evolution paths to 2050. The inevitable trend of ageing will replace the slow growth of the population and become the first macrostate of world population evolution, which indicates that the demographics on all continents are inevitably ageing, at present or in the near future, including the current ‘young’ Africa, and Asia, South America with a ‘demographic dividend’.

## Figures and Tables

**Figure 10.**
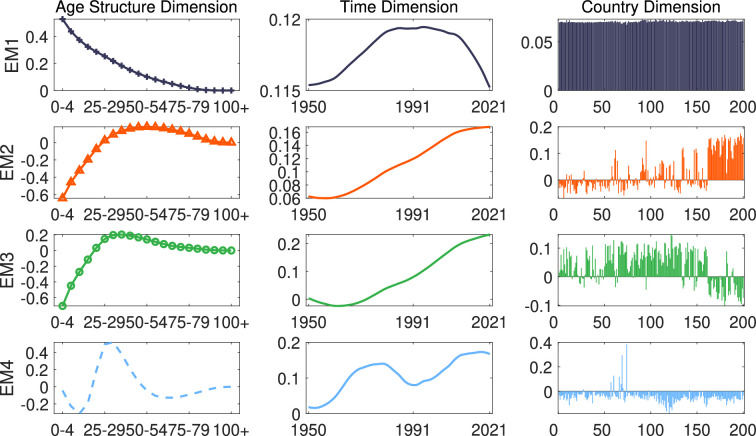
Decomposition results with R=4 .

**Figure 11.**
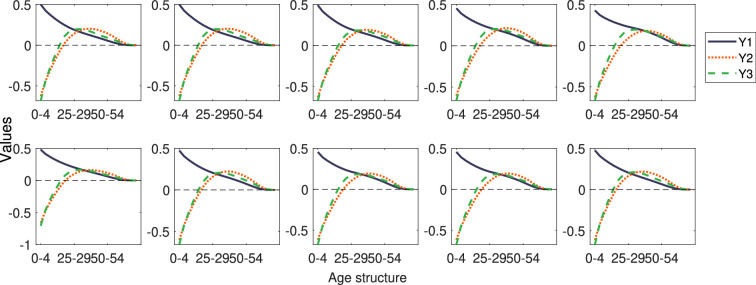
Choose the result with the highest fit values in 10 experiments.

**Figure 12.**
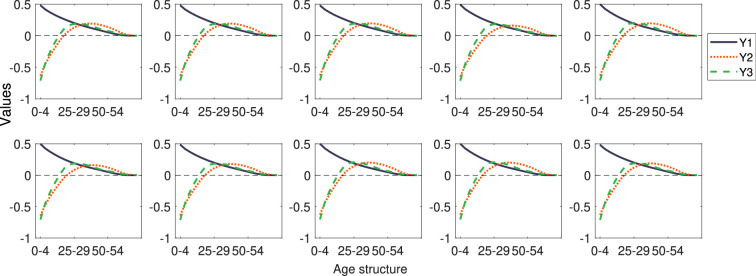
Choose the experiments with the lowest orthogonal indices from the top 10% fit values.

**Figure 13.**
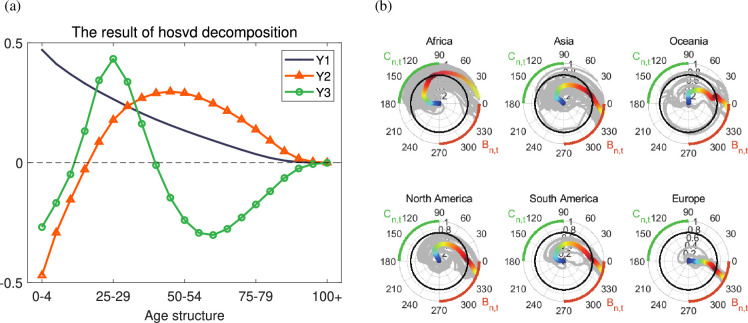
Results of HOSVD decomposition methods. (a) Y1, Y2, and Y3 (corresponding to EM1, EM2 and EM3 in former sessions). (b) Characteristics of age structure evolution.

**Figure 14.**
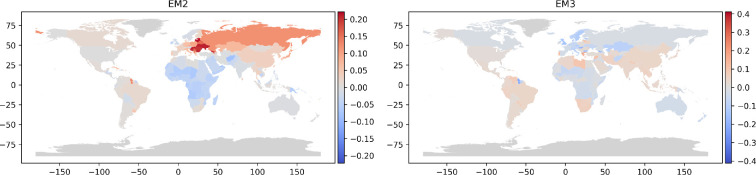
Country dimension of EM2 and EM3.

**Figure 16.**
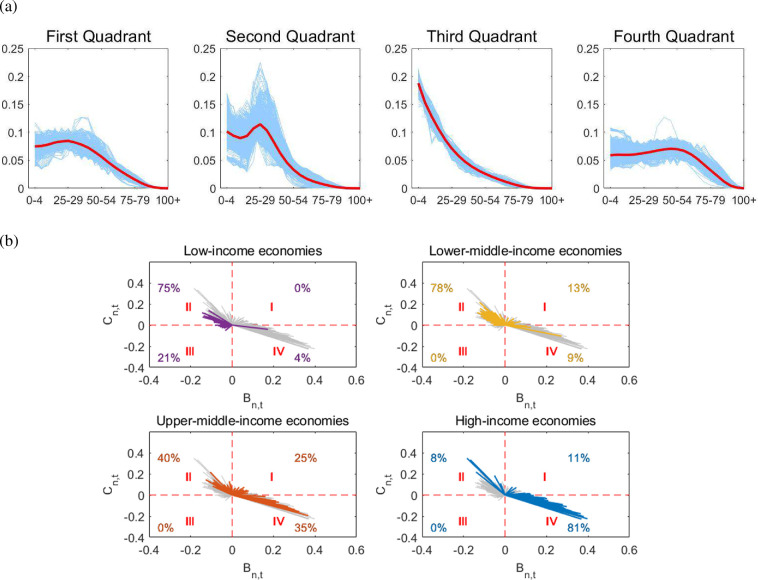
The age structure is represented in the four quadrants. (a) Countries in the four quadrants. (b) Countries of different income levels.

**Figure 17.**
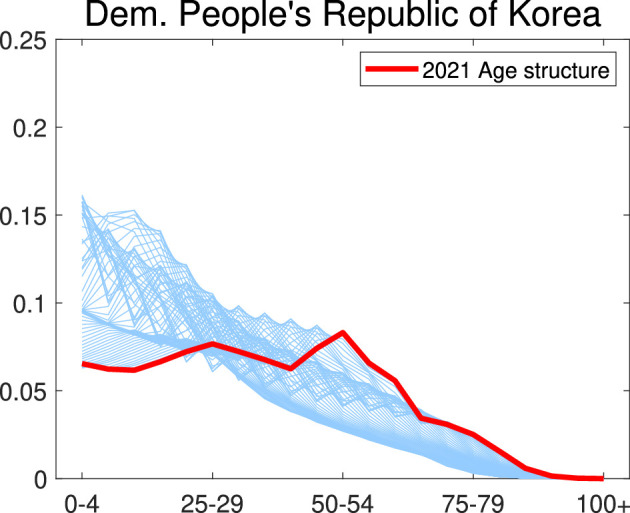
Age structure of PRK.

**Figure 18.**
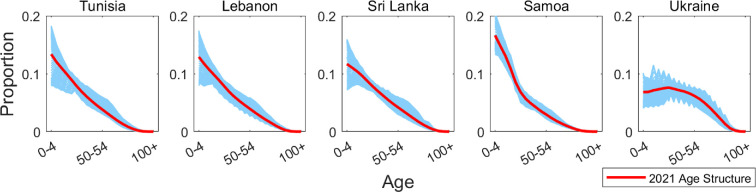
Some lower-middle-income economies.

**Figure 19.**
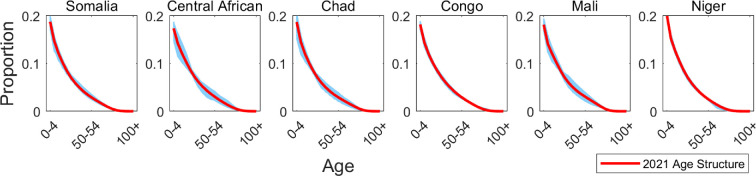
Countries in the third quadrant.

**Figure 20.**
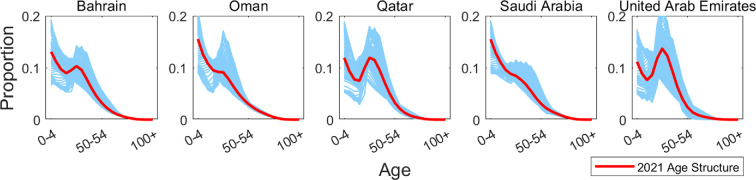
High-income countries in the second quadrant.

**Figure 22.**
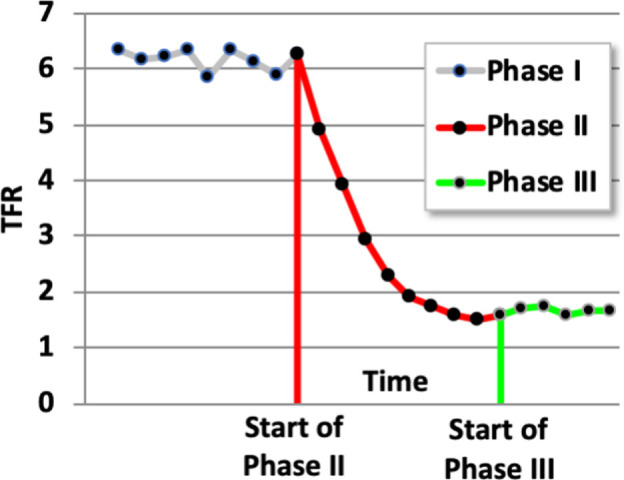
Schematic phases of the fertility transition (live births per woman).

**Figure 23.**
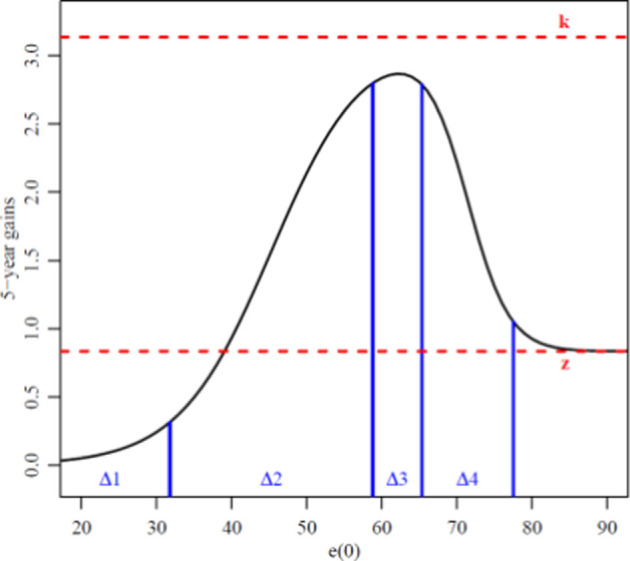
Phases of the mortality transition: gains in life expectancy at birth by level of life expectancy at birth (years).

## Data Availability

The data can be downloaded through the links in table 1.

## Footnotes

^1^
In this paper, ‘country’ and ‘countries’ are used to replace all ‘country/region’ and ‘countries/regions’. This is only a simplification of the text and does not represent the authors’ view on whether different entities are sovereign states.

## Appendix A. Validity of decomposed results

### A.1. Determination of rank as R

For each rank, we randomize the initial value 500 times and choose the final decomposition result according to the fit value. Since the fit values obtained by some random initial values are very close, when we select the optimal result of each rank, in addition to considering the size of the fit value, we also consider the orthogonality index, and hope that each EM explains the information is as varied as possible. The orthogonality index of EM ${,}_{i}$ and EM ${,}_{j}$ can be calculated by inner product, its form is $Z(i,j)=\left|<{𝒂}_{𝒊},{𝒂}_{𝒋}>\right|*\left|<{𝒄}_{𝒊},{𝒄}_{𝒋}>\right|*\left|<{𝒕}_{𝒊},{𝒕}_{𝒋}>\right|$ . The expression to measure the overall orthogonality of the decomposition results is as follows:

(A 1)
$$\begin{array}{lcr} & Z=\sum_{i=1}^{R}\sum_{j=i+1}^{R}\left|<{𝒂}_{𝒊},{𝒂}_{𝒋}>\right|*\left|<{𝒄}_{𝒊},{𝒄}_{𝒋}>\right|*\left|<{𝒕}_{𝒊},{𝒕}_{𝒋}>\right|. & \end{array}$$

The lower the orthogonality index, the higher the orthogonality between EMs in the decomposition results, and the more different interpretation angles of each EM will be. We set the random initial value 500 times under the given rank, and consider the decomposition result with the fit value in the top 10% and the lowest orthogonality index as the optimal one. It is worth noting that this choice will not lose the fit value. The difference between the highest fit value and the fit value considered to be orthogonal does not exceed 1%, and the difference under some ranks is 0.

As shown in figure 9, we drew the fit value f under different ranks. The two curves are the fit value f and the percentage of growth f(n)−f(n−1)/f(n−1) (where n is the rank value, n≥2 ). It shows that when the rank value is greater than 3, a significant increase in the rank does not lead to a significant increase in the fit value.

Furthermore, we plot the decomposition results with R=4 (figure 10). It is consistent with that of R=3 in the first three EMs, highlighting the overall trend, ageing and labour force, respectively, and the values of most countries are more prominent in the country dimension. However, the value of the national dimension of EM4 is reflected in individual countries, and it is not a general rule that most countries have. In summary, we choose R=3 here.

### A.2. The results of 10 experiments

Based on R=3 , we conducted 10 experiments, and each experiment randomly assigned 500 initial values. The decomposition results corresponding to the best-fitting value obtained in each experiment are as follows. Figure 11 shows the age structure of the decomposition results. The first row of table 2 showsthe fitting-values of the 10 experiments in figure 11.

**Table 2.** Fit value and orthogonality index. (*Results from the top 10% fit value experiments with the minimum orthogonal index.)

experiment number	1	2	3	4	5	6	7	8	9	10
maximum fit value	0.8873	0.8873	0.8875	0.8873	0.8874	0.8874	0.8875	0.8874	0.8874	0.8874
corresponding orthogonality index	1.0549	1.0549	1.4753	1.2451	1.3315	1.1832	1.3087	1.4102	1.4102	1.1008
orthogonal—fit values	0.8865	0.8865	0.8864	0.8865	0.8867	0.8865	0.8866	0.8868	0.8868	**0.8865***
corresponding orthogonality index	0.6007	0.6007	0.5812	0.6203	0.6096	0.5972	0.6059	0.6429	0.6429	**0.5780***

We also considered the orthogonality index such that the three age structures obtained from the decomposition results should be as orthogonal as possible, and the second row of table 2 shows the orthogonalindexs of the 10 experiments in figure 11. The following is the results of each experiment with fit value ranking in the top 10% and orthogonal index minimum, figure 12 shows the age structure of the decomposition results, with the fitting-values and orthogonal indexs in the third and fourth rows of table 2. In all cases, the revealed characteristics of age structure were similar. The two major evolutionary trends are as follows: prominent ageing and prominent growth in the labour force. We obtained similar and stable results in these experiments.

In summary, we sacrificed less than 1% (fit value comparison) of the fit value for a result with a low orthogonality index, as the asterisk in table 2.

### A.3. Other decomposition methods

We also used HOSVD and other decomposition methods and found that the age structure of EM1 is still the overall trend [35,37], and the age structures of EM2 and EM3 had maximum values in the elderly and labour ages, respectively. The difference is that the HOSVD decomposition results showed that the maximum age proportion of EM3 was more prominent than that obtained by CP decomposition. However, this will not affect the universal evolution law of the age structure we proposed (as in figure 13).

## Appendix B. Country dimensions for decomposition results

In figure 14, the values of ${𝒄}_{2}$ and ${𝒄}_{3}$ in each country are shown separately. The countries corresponding to the positive value of ${𝒄}_{2}$ are mainly concentrated in Europe (left), and the countries corresponding to the positive value of ${𝒄}_{3}$ are mainly concentrated in Africa, Asia and other regions (right).

## Appendix C. The coefficient of the ageing trend will keep on increasing in the next 20 years

Figure 15 is a supplement to figure 4b with forecast data from the 2020s to the 2040s. Here, the ridgeplot shows the distribution and evolution of $B_{n,t}$ and $C_{n,t}$ . The distributions of $B_{n,t}$ and $C_{n,t}$ gradually evolved from unimodal to bimodal distributions, reflecting the trend of countries’ demographic characteristics from similarly to polarized. For $B_{n,t}$ (red), the peak on the right gradually replaced the peak on the left to become the most frequent one, meaning the ageing trend will continue to increase in the next 20 years, and this phenomenon will occur in most countries.

## Appendix D. Age structure characteristics of representative countries in four quadrants

Figure 16a shows the age structure characteristics of some countries, with blue lines describing the age structures for each country and red lines representing their mean value. With $B_{n,t}$ and $C_{n,t}$ as horizontal and vertical coordinates, the positions of countries fall in the first to fourth quadrants (figure 16b). The second quadrant (II) has the prominent characteristics of the working-age population structure, and most low-income (75%) and lower-middle-income (78%) countries belong to this type. The third quadrant (III) has the prominent characteristics of the underage population structure, including only six low-income countries, and some countries have more than 46.53% of the population under the age of 14. The fourth quadrant (IV) has prominent characteristics of the elderly population structure, including most high-income countries (81%).

## Appendix E. Evolution of age structure in some countries

Democratic People’s Republic of Korea (PRK) is the only low-income country with an ageing trend figure 17 and 18 presents some ageing countries at the low-middle-income level. Here, the blue lines represent the age structure of these countries from 1950 to 2020, and the red line represents their age structure of 2021. The blue lines in these countries all feature an arched proportion of the elderly population, especially in Ukraine, which is most evident.

Figure 19 depicts the only five countries in the third quadrant in 2021 with prominent characteristics of underage population structure, including Somalia (SOM), Central Africa (CAF), Chad (TCD), Democratic Republic of the Congo (COD), Mali (MLI) and Niger (NER).

Figure 20 shows the five high-income countries in the second quadrant with prominent characteristics of the workforce, including Bahrain (BHR), Oman (OMN), Qatar (QAT), Saudi Arabia (SAU) and the United Arab Emirates (ARE).

## Appendix F. Analysing the alternating of the elderly and working-age population

When analysing the alternating of the elderly and working-age population, in figure 5*c*, the evolutionary trends of all countries across various time periods have been meticulously analysed. The original data depicted in the box plot is illustrated by the circular points within figure 21. These points symbolize the proportion of time a country rotates clockwise during the corresponding time period. For instance, if a country rotates clockwise for 72 years during the period from 1950 to 2021 (totalling 72 time periods), the proportion is 1. We calculated the proportion of clockwise rotation of each country in different time periods and found that a clockwise trend was present in the evolution of most countries. Especially since the 1990s, more than 70% of countries have experienced clockwise evolution, accounting for more than 80% of the paths. In the last 10 years, this clockwise ratio has been very close to 100%.

## Appendix G. Methodology of the United Nations population estimates and projections

The United Nations is the main organization that produces regularly updated estimates and forecasts of the population for different countries/regions and its population forecasting track record is relatively good [69]. The 2022 Revision of World Population Prospects is the edition of official United Nations population estimates and projections that have been prepared by the Population Division of the Department of Economic and Social Affairs of the United Nations Secretariat. The preparation of each new revision of the official population estimates and projections of the United Nations involves two distinct processes: (i) the incorporation of new information about the demography of each country or area of the world, involving a reassessment of past estimates where warranted; and (ii) the formulation of detailed assumptions about the future paths of fertility, mortality and international migration for every country or area of the world.

The population estimates and projections contained in this revision cover a 150 year time horizon. Estimates refer to the period from 1 January 1950 to 1 January 2022. This latest assessment considers the results of 1910 national population censuses conducted between 1950 and 2021, as well as information from vital registration systems and from 3189 nationally representative sample surveys, and the projections are for the period from 1 January 2022 to 1 January 2101. For each of 237 countries or areas, the population estimates and projections were produced by starting with a base population by age and sex for 1 January 1950 and advancing the population through successive single-year intervals of time using the cohort-component method for projecting population (CCMPP). The CCMPP relies on information about: fertility by age of mother to determine the number of births taking place each year; mortality by sex and age to determine the number of deaths; and net international migration by sex and age to determine the levels and patterns of population shifts across international borders [1].

CCMPP provides an accounting framework for the three demographic components of population change—fertility, mortality and international migration—and applies it to the population in question such that the ‘demographic balancing equation’ is preserved as [1]:

(G 1)
P(t+n)=P(t)+B(t→t+n)–D(t→t+n)+NM(t→t+n),

where n is the length of the projection interval, P(t) is the population at the beginning of the projection interval, P(t+n) is the population at the end of the projection interval, B(t→t+n) is the births within the projection interval, D(t→t+n) is the deaths within the projection interval and NM(t→t+n) is the net international migration within the projection interval (immigrants less emigrants). Technically, CCMPP is not a complete projection method on its own, since it requires that the components of change be projected in advance. Rather, it is an application of matrix algebra that enables demographers to calculate the effect of assumed future patterns of fertility, mortality and international migration on a population at some given point in the future [70,71]. Next, we will discuss the estimation of different attributes one by one.

### G.1. Estimating total fertility rate (TFR)

Firstly, the preferred source of data on fertility is counts of live births, by age of mother, from a system of civil registration with national coverage and a high level of completeness [72]. In cases where the registration of births is deficient or lacking, fertility estimates are typically obtained through sample surveys.

In the 2022 Revision, Bayesian hierarchical models were used to estimate the annual time series of total fertility rate (TFR), age-specific fertility and the sex ratio at birth from 1950 through 2021 for all countries. These models incorporated available empirical evidence from vital statistics, population censuses and demographic surveys. Here the bayesTFR package for R provides a set of functions to produce probabilistic projections of the total fertility rates for all countries [73], and the package bayesTFR is available from https://CRAN.R-project.org/package=bayesTFR.

Estimation of TFR is based on a three-phase model for the evolution of TFR over time in a country [74]. Phase I: pre-transition phase with fluctuations at high fertility level. Phase II: transition from high to low fertility, where decrements are modelled by a random walk with drift given by a double logistic function. Phase III: post-transition phase where fertility fluctuates around the replacement level (a level close to 2.1), modelled by an autoregressive AR(1) process (figure 22).

The pre-transition phase (phase I) is not modelled, as all countries have already entered phase II. Thus, for the purpose of projecting into the future it is not needed. The fertility transition phase (phase II) is modelled by a random walk with drift. This is specified by

$$\begin{array}{c} f_{c,t+1}=f_{c,t}-d_{c,t}\;\;\text{for}\;\;\tau_{c}\leq t<\lambda_{c}, \end{array}$$

where $f_{c,t+1}$ denotes the TFR in country c and time period t , $\tau_{c}$ denotes the start period of phase II for country c , $\lambda_{c}$ is the start period of phase III for country c , while $g(\theta_{c},f_{c,t})$ and $\theta_{c}$ denote the parametric decline function and the corresponding country-specific parameters, respectively. The decrement $d_{c,t}$ is modelled as the sum of a function of the level of the TFR and the noise, as:

$$\begin{array}{c} d_{c,t}=d(\theta_{c},\lambda_{c},\tau_{c},f_{c,t})=g(\theta_{c},f_{c,t})+\epsilon_{c,t}, \end{array}$$

where $g(\theta_{c},f_{c,t})$ are the double logistic decrements, which are determined by the country-specific parameter vector $\theta_{c}=\left(\Delta_{c1},\Delta_{c2},\Delta_{c3},\Delta_{c4},d_{c}\right)$ and given by

(G 2)
$$\frac{-d_{c}}{1+exp(-\frac{2\ln(p_{1})}{\Delta_{c1}}(f_{c,t}-\sum_{i=1}^{4}\Delta_{ci}+0.5\Delta_{c1}))}+\frac{d_{c}}{1+exp(-\frac{2\ln(p_{2})}{\Delta_{c3}}(f_{c,t}-\Delta_{c4}-0.5\Delta_{c3}))}.$$

The random distortions $\epsilon_{c,t}$ in each period have normal distributions as follows:

$$\begin{array}{c} \epsilon_{c,t}=\left\{ \begin{array}{lcl} N(m_{t},s_{t}^{2}), & \text{for} & t=\tau_{c}\\ N(0,\sigma (f_{c,t}{)}^{2}), & \text{for} & \text{otherwise}. \end{array}\right. \end{array}$$

The quantity $\sigma (f_{c,t})$ is the standard deviation of the distortions during the later periods with

$$\begin{array}{c} \sigma (f_{c,t}=c_{1975}(t)(\sigma_{0}+(f_{c,t}-S)(-aI_{\left[S,\infty \right)}(f_{c,t})+bI_{\left[0,S\right]}(f_{c,t})). \end{array}$$

The constant $c_{1975}(t)$ is added to model the higher error variance of the distortions before 1975.

The TFR in the post-transition phase (phase III) is modelled by a first-order autoregressive time-series model [75] as:

$$\begin{array}{c} f_{c,t+1}∼N(\mu_{c}+\rho_{c}(f_{c,t}-\mu_{c}),\sigma^{2})\;\;\text{for}\;\;t\geq \lambda_{c}, \end{array}$$

where $\mu_{c}$ is the country-specific long-term mean fertility rate, and $\rho_{c}$ is the autoregressive parameter with $\rho_{c}\in \left(0,1\right)$ . In bayesTFR these parameters can be estimated via the Markov chain Monte Carlo (MCMC) method. Alternatively, country-independent values can be pre-defined or estimated by maximum likelihood. The start period of phase II, $\tau_{c}$ , is defined as:

$$\begin{array}{c} \tau_{c}=\left\{ \begin{array}{lcl} \max\;\left\{t:(M_{c}-L_{c,t}<0.5)\right\}, & \text{if} & L_{c,t}>5.5\\ \text{first estimation year}, & & \text{otherwise}. \end{array}\right. \end{array}$$

where $M_{c}$ is the maximum observed TFR outcome in country c , and $L_{c,t}$ denote local maxima. The start period of phase III for country c , $\lambda_{c}$ , is defined as the period where two consecutive increases of TFR below 2 have been observed. More formally,

$$\begin{array}{c} \lambda_{c}=\min\;\left\{t:f_{c,t}>f_{c,t-1},f_{c,t+1}>f_{c,t}\;\text{and}\;f_{c,p}<2\;\text{for}\;p=t-1,t,t+1\right\}. \end{array}$$

The method described above uses observed TFR values as input to estimate the model parameters. In the United Nations context, these input values are taken from the latest revision of the WPP, and it developed a method that assesses the uncertainty around past estimates of the observed TFR values and propagates it into the projections [69], with the observed $y_{c,t,s}$ modelled as:

$$\begin{array}{c} \begin{array}{rl} & y_{c,t,s}|f_{c,t}∼N(f_{c,t}+\delta_{c,s},\rho_{c,s}^{2}),\\ & E[\delta_{c,s}]=x_{c,s}\beta ,\\ & E[\rho_{c,s}]=x_{c,s}\gamma . \end{array} \end{array}$$

The $\delta_{c,s}$ and $\rho_{c,s}$ are country-specific parameters which are estimated by maximum likelihood. Here the features used are the sources of the data and the corresponding estimation methods, but the model allows for any user-specified features. This part is combined with the existing Bayesian hierarchical model implemented in bayesTFR. Here, the past TFRs are considered as unknown, and are part of the parameters to estimate.

The estimation of all country-specific parameters and hyperparameters are conditional on the TFRs, other than the TFRs themselves. To estimate past TFR, the model for phase III is estimated together with the phase II model via an MCMC algorithm [76]. This algorithm is a combination of Gibbs sampling, Metropolis-Hastings (for $\Delta_{c,i}$ in equation G 2 and TFR), and slice sampling steps [77].

### G.2. Projecting the medium mortality scenario

The 2022 Revision used probabilistic methods for projecting life expectancy at birth, which allows us to estimate the rate of improvement in life expectancy for a country using past data from that country, and also taking account of the observed past patterns in all other countries. The probabilistic methods used in the 2022 Revision for projecting life expectancy at birth comprise two separate models. The first model depicts the gradual increase over time in female life expectancy at birth [78]. In this model, the transition from high to low levels of mortality is divided into two phases, each of which is approximated by a logistic function that models the gains in life expectancy (figure 23).

The life expectancy at birth, $l_{c,t+1}$ , for country c , in the next year period, t+1 , is projected to be the life expectancy in the current time period, $l_{c,t}$ , plus the expected gain in life expectancy, $g(l_{c,t})$ as:

$$\begin{array}{c} l_{c,t+1}=l_{c,t}+g(l_{c,t}). \end{array}$$

The expected 5-year gain in life expectancy is a double-logistic function of the current level of life expectancy—namely,

(G 3)
$$\begin{array}{lcr} & \begin{array}{rl} g(l_{c,t}|\theta^{c})= & \frac{k^{c}}{1+\text{exp}(-\frac{A_{1}}{\Delta_{2}^{c}}(l_{c,t}-\Delta_{1}^{c}-A_{2}\Delta_{2}^{c}))}\\ & +\frac{z^{c}-k^{c}}{1+\text{exp}(-\frac{A_{1}}{\Delta_{4}^{c}}(l_{c,t}-\sum_{i=1}^{3}\Delta_{i}^{c}-A_{2}\Delta_{4}^{c}))}. \end{array} & \end{array}$$

$\theta^{c}=\left(\Delta_{1}^{c},\Delta_{2}^{c},\Delta_{3}^{c},\Delta_{4}^{c},k^{c},z^{c}\right)$ are the six parameters of the double-logistic function for country c , whose meaning is illustrated in figure 23. The vector $\theta^{c}$ of the parameters for country c are chosen by a United Nations analyst from the five possibilities $(\theta^{\text{Very slow}},\theta^{\text{Slow}},\theta^{\text{Medium}},\theta^{\text{Fast}},\theta^{\text{Very fast)}}$ . The constants $A_{1}=4.4,A_{2}=0.5$ are chosen so that the parameters $\Delta_{i}^{c}:i=1,2,3,4$ are on an interpretable scale, but they are arbitrary in that they could be changed without altering the results, provided that their product, $A_{1},A_{2}$ , remains unchanged.

After adding a random perturbation and to equation (G 3) and allow the parameters of the double-logistic function to vary between countries, it gets

The spread of the residuals clearly decreases with increasing life expectancy, and it is modelled $\epsilon_{c,t}$ as normally distributed with standard deviation proportional to the regression spline fitted to the absolute residuals.

It adopts a Bayesian approach to estimating our model, making it a Bayesian hierarchical model. This model allows us to pool information about the rates of gains across countries by assuming that each set of country-specific double-logistic parameters is randomly sampled from a common truncated normal distribution. So it requires specifying prior distributions for the 13 world parameters of the model: $\left(\Delta_{i},\sigma_{\Delta_{i}}^{2}\right)$ for i=1,2,3,4 , $k,\sigma_{k}^{2},z,\sigma_{z}^{2}$ and ω . To set the parameters of these priors, it first fits the double-logistic model by least squares to the data from each country individually; then, for each parameter, it computed the empirical average squared deviations from the values for the United Nations medium-pace model. Next, it sets the prior means of the reciprocals of the world variance parameters equal to the reciprocals of these values.

The posterior distribution of the world and country-level parameters was approximated by MCMC implemented in R which could be publicly available as bayesLife [79].

The second model used for projecting future mortality trends addresses the gap between female and male life expectancy at birth. Results obtained using the model of the sex gap in life expectancy were combined with those from the model of female life expectancy in order to derive projections of male life expectancy. The 2022 Revision took the same approach as the 2019 Revision to project the life expectancy at birth of countries affected by the HIV and AIDS epidemic.

### G.3. International migration in the medium scenario

International migration is the component of population change that is most difficult to project. Data on past trends are often sparse or incomplete. Given that migration flows to and from most countries tend to be small relatively to the size of the total population, these errors are not expected to substantially influence the population projections.

The basic approach for formulating future net international migration assumptions is straight-forward for most countries. For any given country, a distinction was made between international migration flows and the movement of refugees. For some countries, the projected sex and age pattern of net international migration is represented by the average distribution estimated over the period 2011–2021, smoothed over age using a simple moving average. For several countries of the Gulf region where demographic change is dominated by the flows of temporary labour migrants, an effort was made to model the return flow of those migrants, taking into account their ageing.

As with estimates, net migration balancing was carried out for projections as a final step to ensure that at the global level the sum of all net international migration flows equalled zero for each year of the projection period.

### G.4. Ten projection scenarios and population projection method

For the projection horizon covering calendar years 2022 through to 2100, the annual series of fertility rates for each country were developed through probabilistic models of total fertility, combined with some assumptions about how patterns of fertility by age of mother shift with changes in the total fertility level. Similarly, the annual series of mortality rates were developed through probabilistic models of life expectancies at birth, by sex, together with assumptions about how age-specific mortality rates change with improved survival. The total fertility and life expectancy models considered the historical levels and trend estimated for each country to give a median projected trajectory, as well as statistical bounds of uncertainty (prediction intervals or PIs), for each of these indicators.

Population projection was carried out using the same CCMPP engine as for estimates. Two types of population projections were included in the 2022 Revision: a deterministic version and a probabilistic version.

In the deterministic versions, the population was projected using the trajectories of fertility, mortality and net international migration described in section G.1, G.2, G.3. Computations for geographical aggregates and other country groupings were carried out as described in section I.A World Population Prospects 2022 [69].

The probabilistic version extended the deterministic medium scenario by incorporating uncertainty about future changes in fertility and mortality. For each country, 2000 population projections were computed from 2022 to 2100, each one using a projected trajectory of total fertility rates and a projected trajectory of life expectancy in the country sampled from the predictive distribution of these quantities. The various population and demographic indicators, including vital events and vital rates, were computed for each trajectory, and summarized through 80% and 95% prediction intervals.

## Appendix H. List of countries

See table 3.

**Table 3.** List of countries/regions.

Africa			Asia			America and Oceania^a^			Europe		
no.	countries/regions	ISO3	no.	countries/regions	ISO3	no.	countries/regions	ISO3	no.	countries/regions	ISO3
1	Burundi	BDI	58	Armenia	ARM	109	Antigua and Barbuda	ATG	160	Belarus	BLR
2	Comoros	COM	59	Azerbaijan	AZE	110	Aruba	ABW	161	Bulgaria	BGR
3	Djibouti	DJI	60	Bahrain	BHR	111	Bahamas	BHS	162	Czechia	CZE
4	Eritrea	ERI	61	Cyprus	CYP	112	Barbados	BRB	163	Hungary	HUN
5	Ethiopia	ETH	62	Georgia	GEO	113	Cuba	CUB	164	Poland	POL
6	Kenya	KEN	63	Iraq	IRQ	114	Curaçao	CUW	165	Republic of Moldova	MDA
7	Madagascar	MDG	64	Israel	ISR	115	Dominican Republic	DOM	166	Romania	ROU
8	Malawi	MWI	65	Jordan	JOR	116	Grenada	GRD	167	Russian Federation	RUS
9	Mauritius	MUS	66	Kuwait	KWT	117	Guadeloupe	GLP	168	Slovakia	SVK
10	Mayotte	MYT	67	Lebanon	LBN	118	Haiti	HTI	169	Ukraine	UKR
11	Mozambique	MOZ	69	Qatar	QAT	119	Jamaica	JAM	170	Denmark	DNK
12	Réunion	REU	68	Oman	OMN	120	Martinique	MTQ	171	Estonia	EST
13	Rwanda	RWA	70	Saudi Arabia	SAU	121	Puerto Rico	PRI	172	Finland	FIN
14	Seychelles	SYC	71	State of Palestine	PSE	122	Saint Lucia	LCA	173	Iceland	ISL
15	Somalia	SOM	72	Syrian Arab Republic	SYR	123	Saint Vincent and the Grenadines	VCT	174	Ireland	IRL
16	South Sudan	SSD	73	Turkey	TUR	124	Trinidad and Tobago	TTO	175	Latvia	LVA
17	Uganda	UGA	74	United Arab Emirates	ARE	125	United States Virgin Islands	VIR	176	Lithuania	LTU
18	United Republic of Tanzania	TZA	75	Yemen	YEM	126	Belize	BLZ	177	Norway	NOR
19	Zambia	ZMB	76	Kazakhstan	KAZ	127	Costa Rica	CRI	178	Sweden	SWE
20	Zimbabwe	ZWE	77	Kyrgyzstan	KGZ	128	El Salvador	SLV	179	UK	GBR
22	Cameroon	CMR	79	Turkmenistan	TKM	130	Honduras	HND	181	Bosnia and Herzegovina	BIH
23	Central African Republic	CAF	80	Uzbekistan	UZB	131	Mexico	MEX	182	Croatia	HRV
24	Chad	TCD	81	Afghanistan	AFG	132	Nicaragua	NIC	183	Greece	GRC
25	Congo	COG	82	Bangladesh	BGD	133	Panama	PAN	184	Italy	ITA
26	Democratic Republic of the Congo	COD	83	Bhutan	BTN	134	Argentina	ARG	185	Malta	MLT
27	Equatorial Guinea	GNQ	84	India	IND	135	Bolivia (Plurinational State of)	BOL	186	Montenegro	MNE
28	Gabon	GAB	85	Iran (Islamic Republic of)	IRN	136	Brazil	BRA	187	North Macedonia	MKD
29	Sao Tome and Principe	STP	86	Maldives	MDV	137	Chile	CHL	188	Portugal	PRT
30	Botswana	BWA	87	Nepal	NPL	138	Colombia	COL	189	Serbia	SRB
31	Eswatini	SWZ	88	Pakistan	PAK	139	Ecuador	ECU	190	Slovenia	SVN
32	Lesotho	LSO	89	Sri Lanka	LKA	140	French Guiana	GUF	191	Spain	ESP
33	Namibia	NAM	90	China	CHN	141	Guyana	GUY	192	Austria	AUT
34	South Africa	ZAF	91	China, Hong Kong SAR	HKG	142	Paraguay	PRY	193	Belgium	BEL
35	Benin	BEN	92	China, Macao SAR	MAC	143	Peru	PER	194	France	FRA
36	Burkina Faso	BFA	93	China, Taiwan Province of China	TWN	144	Suriname	SUR	195	Germany	DEU
37	Cabo Verde	CPV	94	Dem. People’s Republic of Korea	PRK	145	Uruguay	URY	196	Luxembourg	LUX
38	Côte d’Ivoire	CIV	95	Japan	JPN	146	Venezuela (Bolivarian Republic of)	VEN	197	Netherlands	NLD
39	Gambia	GMB	96	Mongolia	MNG	147	Australia	AUS	198	Switzerland	CHE
40	Ghana	GHA	97	Republic of Korea	KOR	148	New Zealand	NZL	199	Canada	CAN
41	Guinea	GIN	98	Brunei Darussalam	BRN	149	Fiji	FJI	200	United States of America	USA
42	Guinea-Bissau	GNB	99	Cambodia	KHM	150	New Caledonia	NCL			
43	Liberia	LBR	100	Indonesia	IDN	151	Papua New Guinea	PNG			
44	Mali	MLI	101	Lao People’s Democratic Republic	LAO	152	Solomon Islands	SLB			
45	Mauritania	MRT	102	Malaysia	MYS	153	Vanuatu	VUT			
46	Niger	NER	103	Myanmar	MMR	154	Guam	GUM			
47	Nigeria	NGA	104	Philippines	PHL	155	Kiribati	KIR			
48	Senegal	SEN	105	Singapore	SGP	156	Micronesia (Fed. States of)	FSM			
49	Sierra Leone	SLE	106	Thailand	THA	157	French Polynesia	PYF			
50	Togo	TGO	107	Timor-Leste	TLS	158	Samoa	WSM			
51	Algeria	DZA	108	Viet Nam	VNM	159	Tonga	TON			
52	Egypt	EGY									
53	Libya	LBY									
54	Morocco	MAR									
55	Sudan	SDN									
56	Tunisia	TUN									
57	Western Sahara	ESH									
^a^No. 109−133: North America; No. 134−146: South America; No. 147−159: Oceania.											
